# Development of Multiple Aortic Mycotic Aneurysms After Cardiac Catheterization

**DOI:** 10.1177/2324709617740907

**Published:** 2017-11-10

**Authors:** Desiree A. Steimer, John J. Squiers, J. Michael DiMaio, Katherine B. Harrington

**Affiliations:** 1 Baylor University Medical Center, Dallas, TX, USA; 2 The Heart Hospital Baylor Plano, Plano, TX, USA

**Keywords:** *Aspergillus*, aortic pseudoaneurysm, aortic surgery, fungal infection, mycotic aneurysm

## Abstract

A 71-year-old male with a past medical history of coronary artery bypass surgery developed multiple, infected pseudoaneurysms of the ascending aorta and aortic root 1 year after cardiac catheterization. He underwent aortic root replacement with a 24-mm homograft. Tissue culture from operative specimens revealed invasive *Aspergillus fumigatus* infection. He was treated with voriconazole for 3 months. After 1 year, he had no recurrence of symptoms, pseudoaneurysm, or fungal infection.

## Introduction

Mycotic aneurysms involving the ascending aorta are rare but occur more frequently in patients that are immunocompromised.^1-3^ Clinical presentation of these patients is highly variable—fever, leukocytosis, cough, chest pain, endocarditis, and embolic events have all been reported in the literature.^1,2,4,5^ Accurate diagnosis can be difficult and is therefore often delayed in these patients, leading to high associated mortality of 88% to 95%.^2,4-7^ However, aggressive surgical debridement and antifungal therapy can improve outcomes when initiated early.^1,2,5,6^ Cases of mycotic aneurysms developing in patients after cardiac surgery have been reported from several weeks and up to 3 years postoperatively.^3,6^ Our case highlights a patient that developed multiple mycotic pseudoaneurysms due to *Aspergillus* infection 1 year after cardiac catheterization.

## Case Report

**Figure 1.**
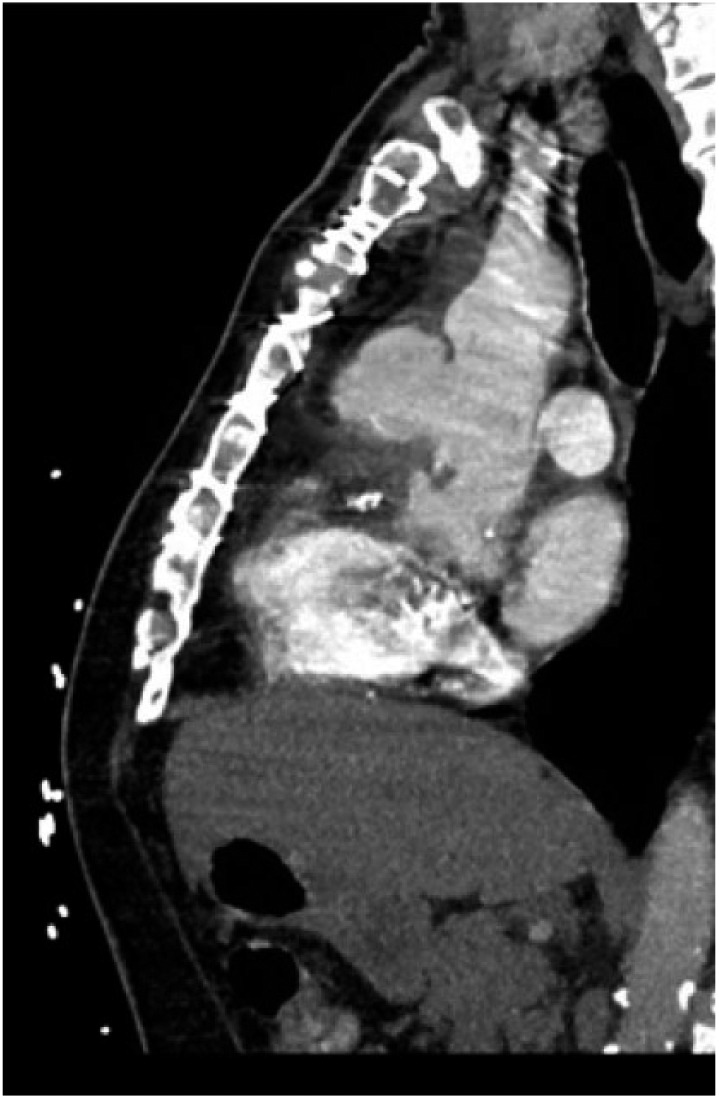
Initial CT angiogram of the chest with aortic pseudoaneurysm.

A 71-year-old male with diabetes, hypertension, and hyperlipidemia underwent coronary bypass surgery (CABG) for 3-vessel coronary artery disease in 2005. He presented 11 years later with chest pain, weight loss, night sweats, and vision changes. He had recently completed treatment for locally advanced prostate cancer with prostatectomy in addition to hormone and radiation therapy. Computed tomography angiography (CTA) of his chest was performed to evaluate for pulmonary embolism and instead revealed an ascending aortic pseudoaneurysm measuring up to 4 cm in diameter (Figure 1). One year prior, he had undergone a cardiac catheterization at another facility, and no pseudoaneurysm was appreciated at that time.

The patient had no leukocytosis or bandemia on presentation nor during his hospitalization, but blood cultures and inflammatory markers were ordered to evaluate for potential infectious etiology. Blood cultures were negative. C-reactive protein was elevated at 4.3 mg/dL. His diabetes was well controlled; HbA1c was 6.5% on admission.

During his workup, both transthoracic and transesophageal echocardiograms were utilized to evaluate the pseudoaneurysm and baseline cardiac function. His left ventricular ejection fraction was 55%. Aneurysmal dimensions measured by echocardiogram were 4.2 × 6.9 cm with extension into the aortic root. Foreign material was floating within the lumen, concerning for an infected pseudoaneurysm. No valvular vegetations were seen. The aortic valve had moderate to severe insufficiency.

Surgical intervention was planned for both the aortic pseudoaneurysm as well as the occluded bypass grafts that had been identified on the recent cardiac catheterization. He was taken to the operating room and the right axillary artery and right femoral vein were cannulated for cardiopulmonary bypass prior to redo-sternotomy. Aorta was cross-clamped distal to pseudoaneurysm and cardioplegia was administered.

**Figure 2.**
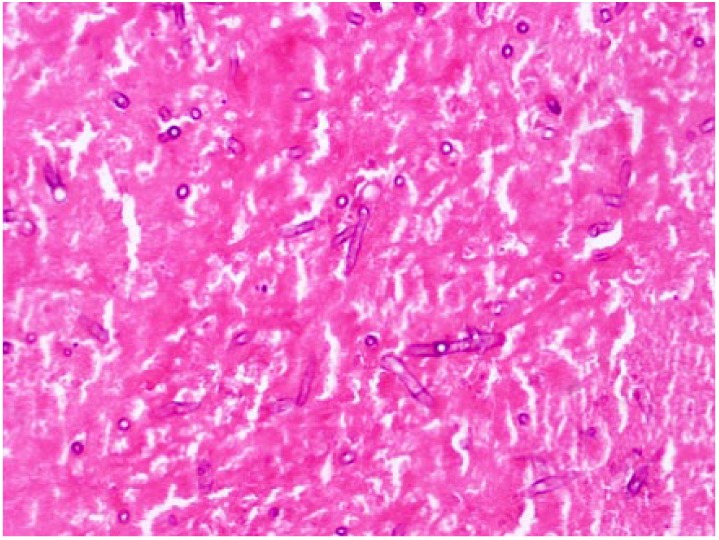
Aortic wall tissue pathology demonstrated invasive *Aspergillus fumigatus* infection (40× magnification).

Contrary to preoperative imaging, the ascending aorta actually had 3 separate pseudoaneurysms. One pseudoaneurysm originated from the aortic root; it had eroded and destroyed the noncoronary aortic valve commissure. The other 2 pseudoaneurysms involved the proximal anastomoses of the coronary bypass grafts. These were both multilobulated and contained several vegetations. Tissue culture was sent from these vegetations, aortic valve leaflets, and the pseudoaneurysm itself. All specimens demonstrated invasive fungal infection with *Aspergillus fumigatus* (Figure 2).

The ascending aorta and aortic root was resected. A 24-mm aortic homograft was selected for aortic root replacement. Due to severe inflammation, the left coronary button was unable to be mobilized and instead was reimplanted using the inclusion technique. The right coronary button was 100% occluded and oversewn. Proximal anastomosis for posterior descending coronary artery and obtuse marginal bypass grafts were implanted in the ascending aortic graft. Mediastinal drains were placed. Axillary and femoral cannulas were removed. Cardiopulmonary bypass time was 320 minutes, and aortic cross-clamp time was 247 minutes.

The morning of surgery, the patient complained of new-onset headaches. Shortly after the procedure, he developed confusion and seizures. CT head without contrast identified a new infarct in the right temporoparietal region. Neurology and infectious disease specialists were consulted due to concern for mycotic embolism. Broad-spectrum antimicrobial therapy was initiated and eventually narrowed to voriconazole after finalized tissue culture results confirmed *Aspergillus fumigatus* infection. The patient’s confusion, short-term memory difficulty, and dysphagia all improved prior to discharge, and he had no long-term neurologic deficits.

**Figure 3.**
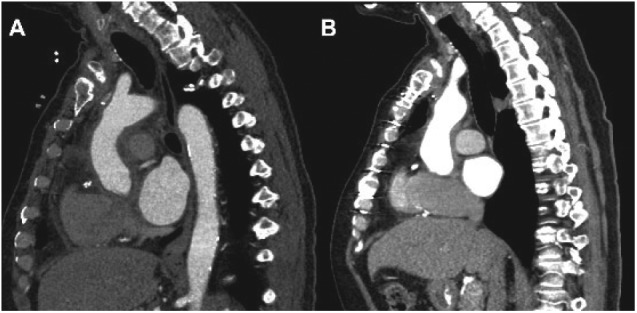
CT angiograms of the chest 1 month (A) and 1 year (B) after intervention.

The patient was discharged on postoperative day 8 to a long-term care facility and eventually returned home. Per infectious disease recommendations, he was treated with long-term voriconazole. Follow-up CTA chest was performed at 1 and 6 months and 1 year postoperatively; no recurrence of pseudoaneurysm was seen (Figure 3). One year after aortic root replacement, he has remained free of recurrent infection and is maintained on voriconazole. The interventional cardiologist who performed the cardiac catheterization prior to the onset of the patient’s symptoms was notified of our findings of a possible procedure-related infection.

## Comment

Although rare occurrences, mycotic aneurysms are known to develop after valve replacement, CABG, or aortic surgery.^2,6^ Current literature suggests that these patients can present weeks, months, or even up to 3 years after their initial surgery.^1,3-6^ There are very few reports of mycotic aneurysm development after cardiac catheterization.^8^ Our patient presented over a decade after CABG with 3 separate mycotic pseudoaneurysms due to *Aspergillus* infection, suggesting the more likely infectious etiology was his cardiac catheterization 1 year prior to presentation. It was discovered at the time of operation that he had 3 separate pseudoaneurysms rather than just the single pseudoaneurysm identified during the preoperative workup. All 3 pseudoaneurysms demonstrated invasive fungal infection.

The exact etiology of these infections remains unclear. Many have speculated that *Aspergillus* spores can infiltrate the ventilation system of the operating room or catheterization laboratory and are subsequently introduced into the sterile field and instruments.^1,2,4,6^ In cardiac procedures that use a graft or valve, the prosthetic material may have been contaminated prior to implantation.^1^ Last, some believe that aortic cannulation for cardiopulmonary bypass is an independent risk factor for these infections because the aortic wall may be weakened during cannulation and therefore becomes more susceptible to infections as well as pseudoaneurysms.^4,6^ Due to the locations of the pseudoaneurysms in our patient, aortic punch for proximal coronary bypass grafts may have increased risk for *Aspergillus* inoculation at the time of catheterization.

In the interval between cardiac catheterization and presentation, the patient was diagnosed and treated for prostate cancer. Although he did not receive chemotherapy, radiation can produce local immunosuppression within the treatment field.^9^ It is possible that his cancer treatment regimen was the inciting event, though this seems unlikely as there are no previous reported cases of distant fungal infection as a result of radiation therapy.

Our case illustrates the importance of maintaining a high index of suspicion when evaluating a patient with a history of prior cardiac surgery or catheterization, as failure to quickly and accurately diagnose aortic aneurysms contributes to a severe mortality rate.^1-6^
